# Differences in hip fracture care in Europe: a systematic review of recent annual reports of hip fracture registries

**DOI:** 10.1007/s00068-021-01797-8

**Published:** 2021-10-08

**Authors:** Maic Werner, Christian Macke, Manfred Gogol, Christian Krettek, Emmanouil Liodakis

**Affiliations:** Trauma Department, Hannover Medical School (MHH), Carl-Neuberg-Str. 1, 30625 Hannover, Germany

**Keywords:** Hip fracture, Registry, Audit, Systematic review

## Abstract

**Purpose:**

Hip fractures are of growing interest due to their increasing number, subsequent functional decline and high institutionalization rate of patients, mortality, and costs. Several process measurements are essential for hip fracture care. To compare and improve these, hip fracture registries in Europe became popular. This systematic review aims to describe the differences between hip fracture registries in Europe as well as the differences in hip fracture treatment between countries.

**Methods:**

A systematic search using the keywords “hip fracture” AND “national” AND “database OR audit OR registry OR register” was performed in PubMed, Embase and Cochrane Library according to PRISMA guidelines till 3rd December 2020. Recent annual reports of identified hip fracture registries in Europe were additionally identified in June 2021. Comparisons of most common case-mix, process and outcome measurements were performed.

**Results:**

11 registries in Europe were identified. Differences were observed regarding inclusion criteria of the different registries. Comparison of the different registries was difficult due to differences in the way to report measurements. While mortality rates differed substantially between countries, most of the process measurements met recommendations according to recent guidelines.

**Conclusion:**

Hip fracture registries were a valid tool to compare hospitals within one country. However, a comparison between registries of different countries should have also been easily possible. For this, the registries need to make their data easily accessible and further unify their way of measuring and reporting.

## Background

Hip fractures are a major health care problem in developed countries. Approximately, 600,000 hip fractures occurred in Europe in 2010 [1]. Calculations expect an ongoing increase with 4.5 million hip fractures worldwide occurring in 2050 [2]. Mostly older patients experience a hip fracture and 25% of them die within 1 year [3, 4].

While the policies in most European countries are partially connected by their membership in the European Union, most aspects of health policies are governed by each country [5]. This results in different health care systems [6]. Disparities in process and outcome measures for hip fracture patients between European countries have been shown [7, 8]. Not only the delivered health care varied, but also the incidence of hip fractures. The highest rate of hip fractures worldwide was occurring in northern Europe, while the lowest rates in Europe were reported in Switzerland and France [9].

Treating hip fracture patients is a challenging task as the high mortality rates are showing. Some process measurements are essential for hip fracture treatment. It could be shown that patients’ mortality rates could be reduced by performing surgery within 24 h of admission to the hospital [10]. Treating these patients together with colleagues in geriatric medicine—so-called orthogeriatric co-management or geriatric trauma unit—showed an additional reduction in mortality rates [11]. Treating osteoporosis reduced the mortality after hip fracture and a falls risk assessment reduced the risk of falling in older adults [12, 13].

To improve the management and outcome of these patients continuously, it is necessary that process and outcome parameters are collected and compared. In 1988, the first hip fracture registry started in Sweden, followed by Scotland in 1993 [14, 15]. Since then, many other hip fracture registries started to collect data, most of them in Europe [16]. However, the variables, which the registries collected, differed between the registries in Europe. To improve the comparability of outcome and process measures in hip fracture registries, the Fragility Fracture Network (FFN) prepared a minimum common dataset in 2013 that is already used in many of them [17].

The purpose of this systematic literature review is to determine the differences in hip fracture care within Europe by comparing the current literature and latest annual reports. Additionally, the differences in the methods of the different registries and their strengths and weaknesses will be discussed.

## Materials and methods

### Search strategy

A systematic search according to PRISMA guidelines using the search terms and Boolean Operators “hip fracture AND national AND (database OR audit OR registry OR register)” in PubMed, Embase and Cochrane Library was performed as of the earliest records till 3rd December 2020 by the first author. The search in PubMed and Embase was limited to the languages English and German. Abstracts and titles were screened for the clear referring to a hip fracture database/audit/registry/register on national basis in European countries. Registries without the clear sole focus on hip fractures were excluded. Analyses of hospital discharge records, national patient databases, administrative claims databases or health insurance data were not considered. Only online available full-text articles with an available abstract were considered.

With the above-described search, the hip fracture registries in Europe that were included in the quantitative comparison were identified. An online web search for the annual reports of the identified registries was performed. No language restriction for the annual reports was applied. Annual reports not in English or German were translated using a web-based translator. Moreover, the websites of the registries were searched for additional information on methods and the registries were contacted if information on methods were missing.

This review was not registered. Ethical approval was given as a waiver by the ethics committee of Hannover Medical School (Nr. 9135_BO_K_2020).

### Quantitative comparison of registries

For the comparison of hip fracture registries the case-mix, process and outcome parameters reported in equal ways in the different annual reports and most recent papers were identified. Categorical variables were pooled into the mostly used categories. Due to information governance, it was not possible to easily access the raw data of the registries. If the used annual reports or paper did not publish these measurements, the papers of Ojeda-Thies et al. and Johansen et al. who used previous annual reports in their analysis, were mainly used [16, 18]. The data are presented in absolute numbers and percentages, as given in the annual reports. No statistical test for comparison was performed.

## Results

### Study selection

**Fig. 1:**
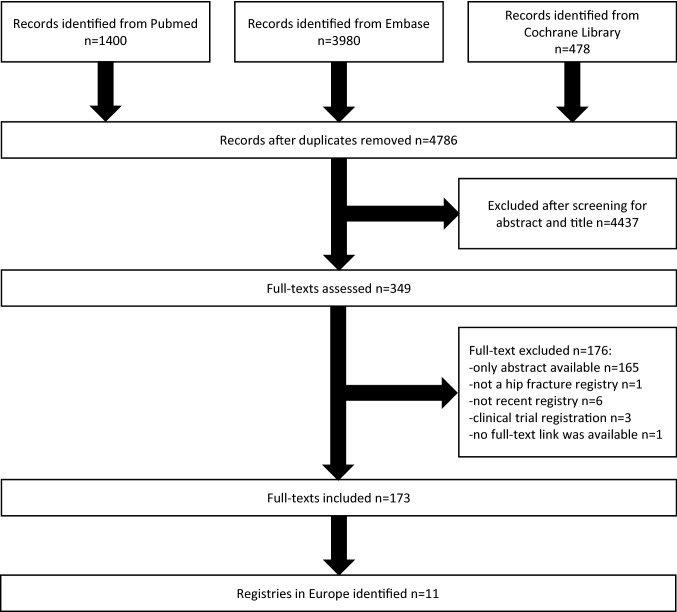
Flow diagram of record assessment

With this search strategy, 3980 records in Embase, 1400 in PubMed and 478 in Cochrane Library till 3rd December 2020 were identified. After removal of duplicates, 4786 records were left. 349 records were assessed as a full-text after discarding the rest based on the abstract and the title. 176 records were excluded further at this step. Mainly because the abstracts had no full-text available as they were often conference abstracts. One record was excluded for not mentioning a hip fracture registry in the full-text, 6 were excluded because the registry mentioned was not up-to-date and a more up-to-date registry for the same country was found, 3 were the abstracts of a clinical trial registration at clinicaltrials.gov and for one article no full-text could be obtained after a thorough search. 173 full-texts were accessed and searched for the in the abstract mentioned hip fracture database/audit/registry/register and the corresponding country. Eleven different countries with a hip fracture database/audit/registry/register were detected. With this web-based search nine annual reports were found: Sweden [14], Scotland [19], Denmark [20], Norway [21], England/Wales/Northern Ireland (National Hip Fracture Database—NHFD) [22], Ireland [23], Germany [24], Netherlands [25] and Spain [26]. For Finland, the data were available in a web-based dashboard [27]. For the NHFD, additional information was also obtained using their dashboard [22]. Italy had no annual report available, only two research papers [28, 29]. The annual reports included for the countries reported in the most cases on the year 2019, but for Finland, the data were presented for the year 2018 and for Italy from 01.02.2016 to 31.07.2018. For some parts of the methods and quantitative analysis, additional paper were used [16, 18, 30–32]. The flowchart of the literature search is displayed in Fig. 1.

### Characteristics of registries

Eleven hip fracture registries in Europe with recent data were identified: four in the north of Europe, two in the Mediterranean area and the rest in central Europe. The first registry started 1988 in Sweden and the latest 2017 in Spain. The registries varied in their inclusion criteria. Most hip fracture registries were including patients with a minimal age of 50 or more. However, the Netherlands included patients as soon as they become adults and Norway tried to include all hip fracture patients, regardless of age. Consent was not necessary in some registries. The inclusion of pathological and periprosthetic fractures was performed differently and not all registries stated this exactly in their inclusion or exclusion criteria, e.g., Germany was the only registry clearly stating in their methodology that they include periprosthetic fractures. Conservative treatment of hip fractures was an exclusion criterion in some registries. In Finland, being a resident of a nursing home was also an exclusion criteria [31]. The follow-up period varied between 1 and 36 months in the different registries. Most importantly, not all registries covered all fractures in their country and hereby only represented a part of the whole patient collective, who were treated in special selected hospitals. The role model for inclusion of most cases was the NHFD with 67,302 cases in 2019 from 174 hospitals in England, Wales and Northern Ireland. In Italy, only 14 hospitals participated and these reported the cases on a voluntary basis. The hospitals that reported data to the German hip fracture registry had to meet special process criteria before they were included in the registry. Not only the inclusion criteria varied extremely between the different registries but also the accessibility of the data. Not all registries published annual reports in English language, which made the correct understanding difficult.

The different inclusion criteria, used from the registries across Europe, were a possible problem, because they could have resulted in a selection bias. Moreover, the interpretations of the data in the annual reports of the registries should be read thoughtfully since the annual reports were created by persons affiliated to the registries.

### Collected variables

To compare hip fracture care between the different countries uniform measurements needed to be used. Many registries aligned their data collection on the minimum common dataset of the FFN [17]. However, some registries differed considerable from this dataset. For example, Norway’s reported data focused more on surgical procedures than on other process or outcome measurements. The Finnish database was retrospectively based on other national databases, which limited the data from this registry. Additionally, the registries presented not all their data in the publicly available annual reports. The way to present these data differed between the registries. Comparison of follow-up data was due to different follow-up timeframes difficult.

### Proportion of registered cases

To interpret the results correctly, data regarding the registered cases was essential. The registries that presented numbers on proportion of the registered cases ranged between 78.9% in Sweden and 100% in Denmark. Danish hospitals were obliged by law to report cases [30]. Finland included all data from the hospital discharge records, and therefore, should have high percentages of registered cases within their inclusion criteria. For Germany, Italy and Spain, no data regarding the proportion of the registered vs. total national cases were reported. In these countries not all hospitals participated and therefore a high percentage of cases in the country were not included (Table 1).

**Table 1:** Registry characteristics

Countries [ref.] variables	Sweden [14]	Scotland [19]	Denmark [20, 30]	Finland [27, 31]	Norway [21]	NHFD [22]	Ireland [23]	Germany [24]	Italy [28, 29]	Netherlands [25, 32]	Spain [26]
Starting year	1988	(1993)	2003	(2004)	2005	(2007)	2012	2016	2016	2016	2017
Age of inclusion (years)	≥ 50	≥ 50	≥ 65	≥ 50	All ages	≥ 60	≥ 60	≥ 70	≥ 65	≥ 18	≥ 75
Consent of the patients necessary	No	No	No	No	Yes	No	No	Yes	Yes	No	Yes
Conservative treatment included	Yes	Yes	No	No	No	Yes	Yes	No	No	Yes	Yes
English language annual report available	Yes	Yes	No	No	Yes	Yes	Yes	No	Only research articles	No	Yes
Follow-up period (months)	4	2	1, 24	1, 3, 6	4, 12, 36	4	1	4	1, 4	3, 12	1
Cases included (n)	12,548	7364	6373	4530	7877	67,302	3701	8231	3017	15,352	13,181
Cases registered of all cases in the country (%)	78.9	99.9^a^	[100]		88–94 (2017–8)	[91]	99			88	
Participating hospitals	45/53	19	21/21	(24/24)	44/44	174/174	16/16	91	14	66/81	80

NHFD: England/Wales/Northern Ireland

^a^Of the participating hospitals

When recent data were not available, data from Ojeda-Thies et al. [16] or Johansen et al. [18] were used indicated by () or []

### Basic patients’ variables

The average patient was older than 80 years and in over 66% female. Prior to fracture, most patients lived at home ranging from 60% in the Netherlands to about 90% in Italy. 33–50% of the patients could walk without an aid outdoors before fracture. Sweden and Spain reported the mobility in different categories as the other registries and were, therefore, not included in the table and Denmark used the Cumulated Ambulation Score (CAS). The average patient had many comorbidities reflected by an ASA-Score (American Society of Anesthesiologists risk classification) of ≥ 3 in 58%–74.5% patients. Denmark used for the comorbidities instead of the ASA-Score the Charlson Comorbidity Index (CCI). Also, between 17 and 43.9 percent of the patients had already cognitive problems (Table 2).

**Table 2:** Basic patient variables at admission

Countries Variables	Sweden	Scotland	Denmark	Finland	Norway	NHFD	Ireland	Germany	Italy	Netherlands	Spain	Range
Sex (women) (%)	66	(73)	67.9^a^		69	[72]	69	72	77	67	76.1	66–77
Age (years)												
Mean	82				80	[83]	80	84.3			86.8	80–86.8
Median		(82)	83^a^				81	84	86	81		81–86
Place of residence (%)												
At home	(71)	(75)	74^a^			(81)	85	75	91.6	(60)	(75)	60–91.6
Nursing home	(24)	(18)	17.8^a^			(19)	10	22	8.4	(19)	(24)	8.4–24
Acute hospital	(3)	(6)				3.7	5	1			(0.4)	0.4–6
Mobility status (%)												
Outdoor without aids		50^b^	CAS was used			36.4^b^	46^b^	33	44.4	37^b^		33–50
Outdoor with aids		39^b^				36.7^b^		48	24.6	31^b^		24.6–48
Only walking indoor		10^b^				23.7^b^	14^b^	14	28.3	6^b^		6–28.3
No walking		1.4^b^				1.7^b^	2^b^	3	2.7	2^b^		1.4–3
cognitive dysfunction (%)^c^	(30)	(26)			26.9	[37]	25		32.2	(17)	43.9	17–43.9
ASA-Score 1–2 (%)	(39)	[26]	CCI was used		34.7	[27]	39	24	25.5	(42)	(29)	24–42
ASA-Score ≥ 3 (%)	(61)	[68]			64.2	[68]	61	74	74.5	(58)	(71)	58–74.5
Fracture type (%)												
Intracapsular	52	[53]	52.9^a^		60.4	(58.6)	50	44	48.7	55	38.2	38.2–60.4
Pertrochanteric^d^	41	[38]	39.6^a^		33.1	(34.8)	36	44	38.7	39	51.8	33.1–51.8
Subtrochanteric	8	[4]	7.5^a^		5.1	(5.9)	7	4	8.6	4	8.8	4–8.8
Other		[5]			1.0		4	8	4	3		1.0–8

NHFD: England/Wales/Northern Ireland; CAS: Cumulated Ambulation Score; ASA-Score: American Society of Anesthesiologists risk classification; CCI: Charlson Comorbidity Index

^a^Data from 2018 [30]

^b^Data from Voeten et al.[32]

^c^Cognitive dysfunction: Short Portable Mental Status Questionnaire (SPMSQ): Spain > 3 errors and Italy > 4 errors; Abbreviated Mental Test Score (AMTS): NHFD > 2 errors and Ireland > 3 errors

^d^Basocervical fractures if separately reported

When recent data were not available, data from Ojeda-Thies et al. [16] or Johansen et al. [18] were used indicated by () or []

### Process variables

The operative treatment varied with the predominant fracture type between a hemiarthroplasty and a femoral nail. A dynamic hip screw (DHS) was used in Germany, Spain and Italy very rarely in comparison to other countries. Most of the prostheses were cemented. The anaesthetic procedure varied strongly between the different countries, too. While in Germany spinal anaesthesia was not common, this was the mostly used technique in Sweden and several other countries (Table 3).

**Table 3:** Operative and anaesthetic treatment

Countries Variables	Sweden	Scotland	Denmark	Finland	Norway	NHFD	Ireland	Germany	Italy	Netherlands	Spain	Range
Surgical procedure (%)												
Conservative	(1)					(2.2)	(5)			3	2.4	1–5
Cannulated screws	(15)	(2)	[10]		11.4	(3)	2^a^	(2)	4.2	5	2.2^a^	2–15
DHS	(20)	(34)	[22]		15.3	(32)	15^a^	(3)	4.3	13	1.4^a^	1.4–34
IM-Nail	(27)	(10)	[31]		23.4	(12)	28^a^	(50)	49.9	39	60.5^a^	10–60.5
Hemiarthroplasty	(25)	(48)	[25]		41.7	(43)	47^a^	(34)	24.2	33	33.2^a^	24.2–48
Total hip replacement	(10)	(6)	[10]		7.8	(8)	4^a^	(6)	15.4	7	2.7^a^	2.7–15.4
Cemented prosthesis	[97]	94.7^b^			93.1^b^	92.3	76					76–97
Anaesthetic technique (%)^c^												
Spinal	[95]	[50]			79.6	45.2	77	6	76.7	63	93.1	6–95
General	[5]	[44]			16.0	56.5	24	94	20.2	43	6.3	5–94

NHFD: England/Wales/Northern Ireland; DHS: dynamic hip screw; IM-Nail: intramedullary nail

^a^Of all operations (excluding conservative treatment)

^b^Of the hemiarthroplasties

^c^More than one technique possible per patient

When recent data were not available, data from Ojeda-Thies et al. [16] or Johansen et al. [18] were used indicated by () or []

Only some reports analysed the time from the emergency room until transfer to a ward or operation room. While in Ireland only 25% of the patients were admitted to a ward in under 4 h, this was the case for 81% in Scotland. The longest time frame to surgery was reported from Spain and Italy with a mean of 64.6 h in Spain and 54 h in Italy. In contrast to these long periods till fracture treatment Sweden, Denmark, Germany and the Netherlands showed the highest rates of surgical repair within 24 h with about two third of the patients. The lowest median was reported from Germany with 17.8 h. Mobilization rates on the first day after surgery varied between 68% in Scotland and 82% in Ireland. Joint care with geriatricians was lowest in Netherlands with 74% and highest in the NHFD with 91%. Falls assessment was performed on 83–96.6% of the patients in the reporting registries and bone health assessment was assessed in over 90% of the patients. Falls assessment included in the most registries a review of previous falls, cause of index fall and further risk factors for falling and injury. Bone health assessment was defined in the registries as getting medication for osteoporosis, being assessed for eligibility of medication for osteoporosis or initiated outpatient diagnostics. Different rates of bone health assessment resulted in different rates of medical treatment of osteoporosis after hip fracture ranging from 10% in Germany to 71% in Ireland (Table 4).

**Table 4:** Process measurements

Countries Variables	Sweden	Scotland	Denmark	Finland	Norway	NHFD	Ireland	Germany	Italy	Netherlands	Spain	Range
Time to ward < 4 h (%)		80.8				28.7	25					25–80.8
Time to surgery (hours)												
Mean	23.5				24	34		24.2	(54)		64.6	23.5–64.6
Median					21		26	17.8	41	20		17.8–41
< 24 h (%)	66		69.7		50.3		43	72		67		43–72
< 36 h (%)	86	76.6	86.3			(70.2)	60	83				60–86.3
< 48 h (%)	94			94.7	83.8		76	91	64.7	93	48.1	48.1–94.7
Mobilization 1st day post-surgery (%)		67.7	76^a^			81	82	79			69.9	67.7–82
Orthogeriatric co-management (%)		85^b^				91^b^	82	86	90.2	74	89.6	74–91
Falls assessment (%)		[88]	89.1			96.6	83					83–96.6
Bone health assessment (%)		91.4	90.3			96.5	94					90.3–96.5
Bone protection medication (%)												
Before fracture						7.2	16	(3.9)	4.4	(10)	5.9	3.9–16
At discharge		50[50]	(50)			55.5	71	(10)	31.1	(19)	42.2	10–71

NHFD: England/Wales/Northern Ireland

^a^Within 24 h after surgery

^b^Within 3 days after presentation

When recent data were not available, data from Ojeda-Thies et al. [16] or Johansen et al. [18] were used indicated by () or []

### Outcome variables

The registries reported extreme different lengths of hospital stay from 4.2 days in Finland to 19.5 days in Ireland. The two non-surgical complications presented by some registries were pressure sores and delirium. The percentage varied between 3 and 4.8% in the reporting countries for pressure sores and 25%–30% for delirium. Most patients were discharged in a rehabilitation facility or directly home. The mortality rates in the hospital episode differed considerable between the countries with 1.5% in Italy and 6% in Germany.

Follow-up results were reported heterogeneously. Readmissions were reported only for orthopaedic reasons or for all causes and the time frames varied between 14 and 120 days. So, comparisons were not applicable. The same problem arose for re-operation rates. 2% were re-operated within 30 days and 3%–4% within 120 days. After 2 years, between 3 and 12% were re-operated depending on the fracture type and surgical procedure. The mortality rates after hip fracture varied between 5.5% and 9.5% after 1 month (Table 5).

**Table 5:** Outcome variables

Countries Variables	Sweden	Scotland	Denmark	Finland	Norway	NHFD	Ireland	Germany	Italy	Netherlands		Spain	Range
New developed pressure sores (%)						3.4^a^	3	3.2	4			4.8	3–4.8
Delirium (%)						30.2^a^			24.4^b^				24.4–30.2
Length of stay (days)													
Mean	7.2		[9]	4.2		15.3	19.5	17.0^c^	(11)			9.8	4.2–19.5
Median	6	9	[8]				12	16.0^c^	9	5			5 –16
Discharge location (%)													
Home		(31)				(52)	24	25	12.1	(22)	(37)		12.1–52
Nursing home		(15)				(12)	18	27	10.5	(22)	(32)		10.5–32
Other acute hospital								2	2.8				2–2.8
Rehabilitation		(44)				(17)	44	39	74.6	(33)	(25)		17–74.6
Death in hospital	[4]	(5)	[3]				5	6	1.5		4.9		1.5–6
Readmission (%) {follow-up time (days)}		6.7 {14}^d^	14.1 {30}^d^	13.2 {30}^(d)^				5 {120}^e^			6.3 {30}^d^		
Reoperation (%) {follow-up time (days)}			3.2–12.3 {730}^f^			(3.1) {120}	2 {30}	4 {120}			2.2 {30}		
Mortality during follow-up (%) {follow-up time (days)}	15.1–20.2 {120}^g^	7.7 {30}	9.5 {30}, 27.9 {365}	5.5 {30}, 11.2 {90}, 15 {180}		6.5 {30}		9 {120}			8.3 {30}		5.5–9.5 {30}

NHFD: England/Wales/Northern Ireland

^a^Reported as a contrary statement

^b^On the first post-OP day

^c^Calculated for the living

^d^All cause readmission

^e^Orthopaedic cause readmission

^f^Depending on the fracture type and surgical treatment; data from patients operated in 2017

^g^Depending on gender

When recent data were not available, data from Ojeda-Thies et al. [16] or Johansen et al. [18] were used indicated by () or []

## Discussion

Our systematic review revealed 11 hip fracture registries within Europe. Different inclusion criteria (e.g. age) were detected. Huge variations between the registries were visible with regard to surgical or anaesthetic method. Time to surgery as a key performance measurement varied considerable. Mortality as the most important outcome measurement ranged between 5.5% and 9.5% at 30 days. Several aspects needed to be kept in mind when comparing the different databases.

Errors in different hip fracture databases are known [33–36]. In most cases, the data for the registries were reported by the regular staff and had to be done additionally to their work. In order for a high participation rate, the staff should have time for collecting registry data [37]. Data errors could be reduced using electronic health records or further using data managers [33, 35].

Not all countries included all cases and all hospitals. In Germany about 169.000 hip fractures were counted in 2019 and the German registry reported only 8231 (~ 5%) cases [38]. Therefore, a negative or positive selection bias was possible. The same applied for Italy and Spain.

Moreover, measurements, the way to collect them and also the way to present them, varied between different registries. Not always were all collected measurements presented in the latest annual report. As a result, the comparison between the countries was limited.

The basic patient variables varied depending on the different inclusion criteria, e.g., the inclusion of younger hip fracture patients in the registries could have resulted in lower ASA-Scores [39]. While men are younger when they suffer from a hip fracture, a bigger proportion of male patients could have been expected [3]. Differences in the diagnosis of cognitive impairment might have also been attributed to different inclusion ages. Two tests were mostly used for cognitive dysfunction: the Pfeiffer Short Portable Mental Status Questionnaire (SPMSQ) and the Abbreviated Mental Test Score (AMTS). In addition to different questionnaires used in the registries, the cut-offs varied additionally and comparison was difficult. The walking ability prior to fracture was reported in the registries with different categories or scores. Only Denmark used the Cumulated Ambulation Score (CAS). To reach more comparability Voeten et al. recommended to use common categories like the Fracture Mobility Score that was already used in many registries [40]. Scores like the Parker Mobility Score would need more questions. The aim for registry questionnaires should be simplicity to achieve a good data completeness [40]. Differences in fracture type might have been explained in part with more intertrochanteric fractures occurring in older patients as observed in Spain and Germany [41, 42]. Spain and Germany had the oldest mean age of all registries and also the highest rate of pertrochanteric fractures. However, Italy with a comparable median age had predominantly intracapsular fractures.

Surgical treatment varied between the different countries. This might have been partly explained by different frequencies of fracture types and subtypes. However, there is still ongoing discussion when to use which implant for intertrochanteric or intracapsular fractures [43–46]. Different guidelines in the countries and surgical tradition might have also explained different surgical procedures. Data from the Norwegian Hip Fracture Registry recommended using cemented hemiarthroplasties for femoral neck fracture, because of lower reoperation rates and no differences in 1-year mortality in comparison to uncemented hemiarthroplasty [47]. However, a higher mortality rate within 48 h was found in a recent meta-analysis for cemented hemiarthroplasties, but also not after one year [48].

The same applied for procedures in anaesthesia. While most countries favoured regional anaesthetic techniques over general anaesthesia, yet there seems to be no clear evidence favouring one of both techniques [49–51]. However, the results of a randomised controlled multi-centre study in Germany, focussing on a geriatric population, may show the benefit of spinal or general anaesthesia [52].

The Blue Book, published 2007 by the British Orthopaedic Association and British Geriatric Society, focused on 6 process variables that needed to be improved for good hip fracture care: time to ward < 4 h, time to surgery < 48 h, pressure sore prevention, orthogeriatric co-management, bone health and falls assessment [53]. These variables were at least partly measured in the most registries and reported in their annual reports.

The Blue Book demanded for a surgical repair of hip fractures within 48 h [53]. However, newer analysis came to the conclusion that a time to surgery within 24 h could be more beneficial for patients [10]. Many countries were able to operate about 90% within 48 h. The highest rate of operations within 24 h was seen in Germany with 72%. In Spain and Italy, these numbers were worse and improvement was needed. Spain was already in the process of reducing their time to surgery and reduced the time to surgery about 10 h in comparison with the annual report of 2017 [16].

However, despite this evidence, some authors believe that the benefits of a reduced time to surgery with regard to mortality are explained by a selection bias [54, 55]. A recent study using data of the German registry found also no evidence for a reduction in mortality [56]. This study is one of many examples for important research questions that were more clarified by the use of data from such big databases.

Orthogeriatric co-management for older hip fracture patients has been proven effective in the last years [11]. The best way to perform this co-management is not yet examined according to recent meta-analyses [57, 58]. The frequency of orthogeriatric co-management should be higher than one visit per week, since this model did not resulted in improved mortality or complication rates [59]. Given the positive effect described above, it should be the aim that every geriatric hip fracture patient is managed collaboratively with a geriatrician. In Germany, orthogeriatric co-management was mandatory for the registration in the hip fracture registry. Therefore, the number in whole Germany could be lower. In countries with low inclusion ages, the percentage of patients without the need of orthogeriatric management could influence this comparison.

It was pleasing that nearly all registries that reported on bone health assessment and falls prevention performed them very frequent to prevent further fractures. Germany did not present frequencies on bone health assessment, but the patients with therapy of osteoporosis at discharge were extreme low. This indicated that more patients should have been assessed for bone health during the hospital stay and more patients should have been prescribed medication for osteoporosis.

The reports on outcome variables were rare. Especially delirium is a common complication after hip fracture [60]. Only two registries reported data on delirium and these rates were with about 25% much lower than reported in other studies [60]. As a conclusion, more tests on delirium should be performed with a special focus on the hypoactive form which is much harder to detect and likely underrepresented [28, 61].

The length of stay between the different countries varied extremely. When a rehabilitation unit was an integrated part of the hip fracture ward it was clear that the length of stay was longer. Structural differences between hospitals and countries might have also resulted in different discharge destinations.

A striking point were the reported inpatient mortality rates and mortality rates after 1 month between the registries. The lowest inpatient mortality was reported in Italy with only 1.5%. Germany had the highest with 6%. For the one month mortality the rates differed between 5.5% in Finland and 9.5% in Denmark. Differences in mortality might have been partly explained by different inclusion criteria, e.g. age, and hereby healthier patients. This explanation did not apply to Italy with 75% showing an ASA-Score of 3 or more. Due to the fact that in Italy not all cases in the hospitals had to be reported it might have been that patients who died were less likely included. The low 30-day mortality in Finland was most likely explained by the exclusion of institutionalised patients since institutionalisation is one risk factor for mortality [4]. The high risk of dying in Germany might have been partly explained by their inclusion of periprosthetic fractures, as they are of increased risk of dying [62] Other follow-up measurements were hard to compare due to much variation in collected data and their follow-up period.

Hip fracture registries require funding due to high costs. In the NHFD about 37 Great Britain Pounds (GBP) were needed for central and local data management [63]. These costs were very low in contrast to the hospital costs in the first year after fracture with about 14.000 GBP per hip fracture in the UK [64]. To justify these costs, the effect on improved care must be clear. However, the effect of hip fracture registries on performance improvement is difficult to measure. It is hard to determine which effects can be addressed to continuous performance surveillance and which are rather explained by other improvements in hip fracture care, such as hip fracture care pathways or special dedicated timeslots in the operation theatre. Moreover, the data of a registry seem to be biased for measuring itself improvement. Therefore, Neuburger et al. compared the proportion of patients receiving early surgery and 30-day mortality for all eligible patients in England 4 years prior to the implementation of the NHFD and 4 years after with external data [65]. They found a significant improvement in both outcomes after NHFD implementation. Other reports from Norway or Denmark showed also a performance improvement in some of the measured parameters over the years of implementation [30, 66] Another question may be how often audits and data collection are necessary for continuous improvements. Ferguson et al. found that in Scotland, after stopping yearly audits for 5 years, the improved quality of care declined again [15]. They recommended regular audits for maintaining the achieved improvements.

Quality improvement is not only achieved by comparing process variables within one country, but also by accompanied research. Edwards et al. showed that small improvements in hip fracture care between two groups need high numbers of included patients to be significant [67]. National registries included a large amount of patient information. With big data studies, research questions could be answered more certain and bias would be less of a problem [68].

Hip fracture registries were a valid tool for comparison of important process and outcome markers between hospitals in one country. Possibilities for improvement could be easily identified. However, a comparison between different hip fracture registries was difficult. Not only differences were observed with regard to which data were collected, but also how to present them. Initiatives as the FFN with a common dataset could further improve the comparability between different hip fracture registries. In the era of globalization, it should have been a standard that English language annual reports were online available so that not only hospitals in one country could compare their performance but also whole countries between each other. Sweden provided an English version after contacting and Spain will provide an English version later in the year.

Our study has several limitations. The biggest limitation is that we could not get access to the raw data and analysis was limited to published data. Comparison of data was complicated as described above. Especially the different inclusion criteria, used by the different registries, resulted in a possible selection bias. This limited the comparability of different outcome parameters considerable, as already discussed. With regard to eliminating possible bias, high-quality randomised controlled trials are still superior. Furthermore, registry data could contain errors, as mentioned earlier. Additionally, the institution of the authors of this review is part of the German registry. This should be kept in mind while interpreting the authors’ conclusions.

Besides data limitation, this study has important strengths. To the best of our knowledge, this is the first study that focused on the comparison of only European hip fracture registries. With this concentration on the European registries, a more detailed comparison was possible. Moreover, recent available data were used in this study and together with older data, most aspects of hip fracture care could be compared adequately.

## Conclusion

Hip fracture care varied between the European countries. While hip fracture registries were a good tool to compare hospitals within one country, comparison between different countries and registries was difficult and, therefore, with limitations. Most countries were on the right track for fulfilling the process parameters mentioned in the Blue Book and recent research demanded. Especially, the time to surgery needed to be improved in some countries. The registries should try to make their collected and presented data further uniform, so that international comparisons become more feasible.

## Data Availability

All data analysed in this review are included in this manuscript.

## Abbreviations

AMTS: American Society of Anesthesiologists risk classification
ASA-Score: Abbreviated Mental Test Score
CAS: Cumulated Ambulation Score
CCI: Charlson Comorbidity Index
DHS: Dynamic hip screw
FFN: Fragility Fracture Network
GBP: Great Britain Pound
IM-Nail: Intramedullary nail
NHFD: National Hip Fracture Database
SPMSQ: Short Portable Mental Status Questionnaire
